# Supplementary material for: Freeze-on limits bed strength beneath sliding glaciers
Source: Nat Commun. 2018 Aug 13;9:3242. doi: 10.1038/s41467-018-05716-1 (PMC6089977; doi:10.1038/s41467-018-05716-1)
Supplement: Supplementary file 1 — Supplementary Information [file 41467_2018_5716_MOESM1_ESM.pdf]

Supplementary information for “Freeze-on limits bed strength beneath sliding glaciers”

Colin R. Meyer et al.

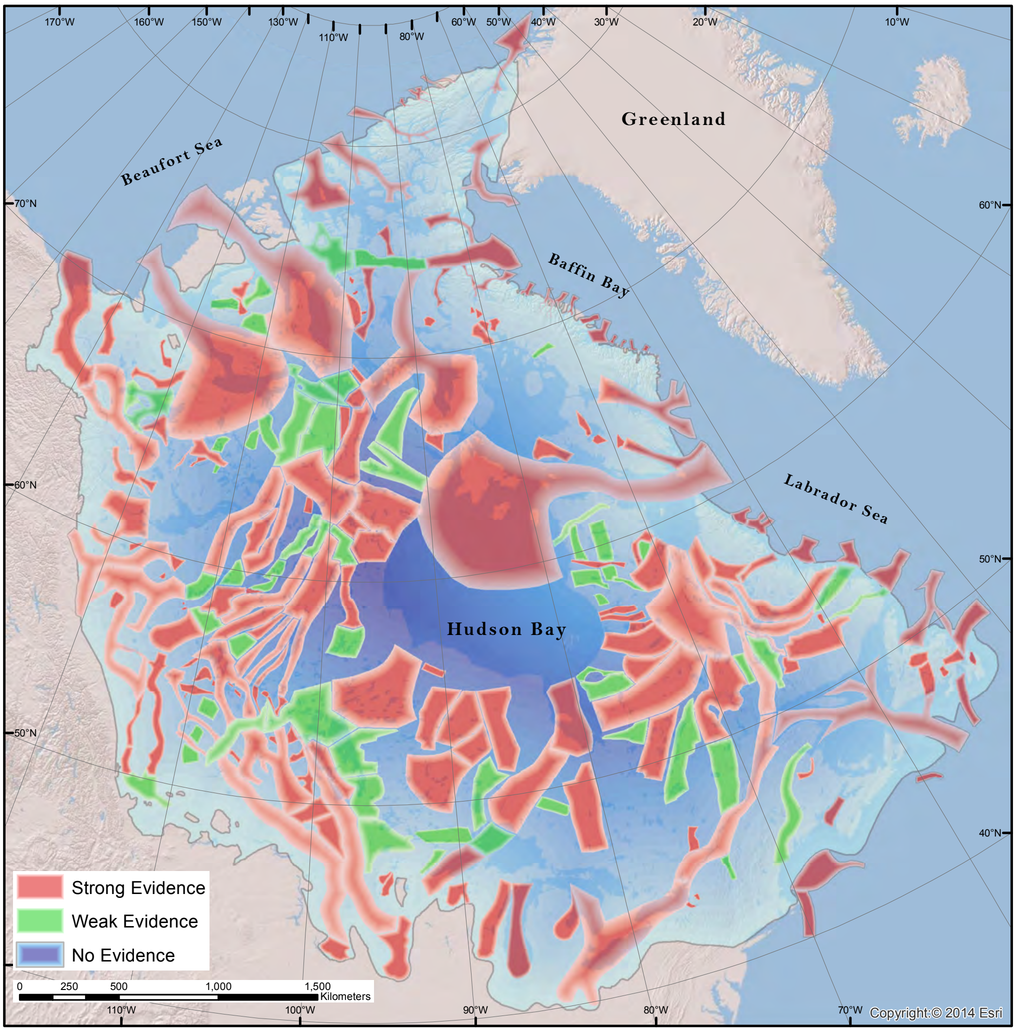

Supplementary Figure 1: Summary figure showing regions with differing degrees of evidence for substantial sliding beneath the last glacial maximum extent of the Laurentide Ice Sheet. Of the 12.85 million km^2 of bed area at the last glacial maximum, 41.7% is characterized by strong evidence for sliding and 8.4% has weak evidence for sliding.

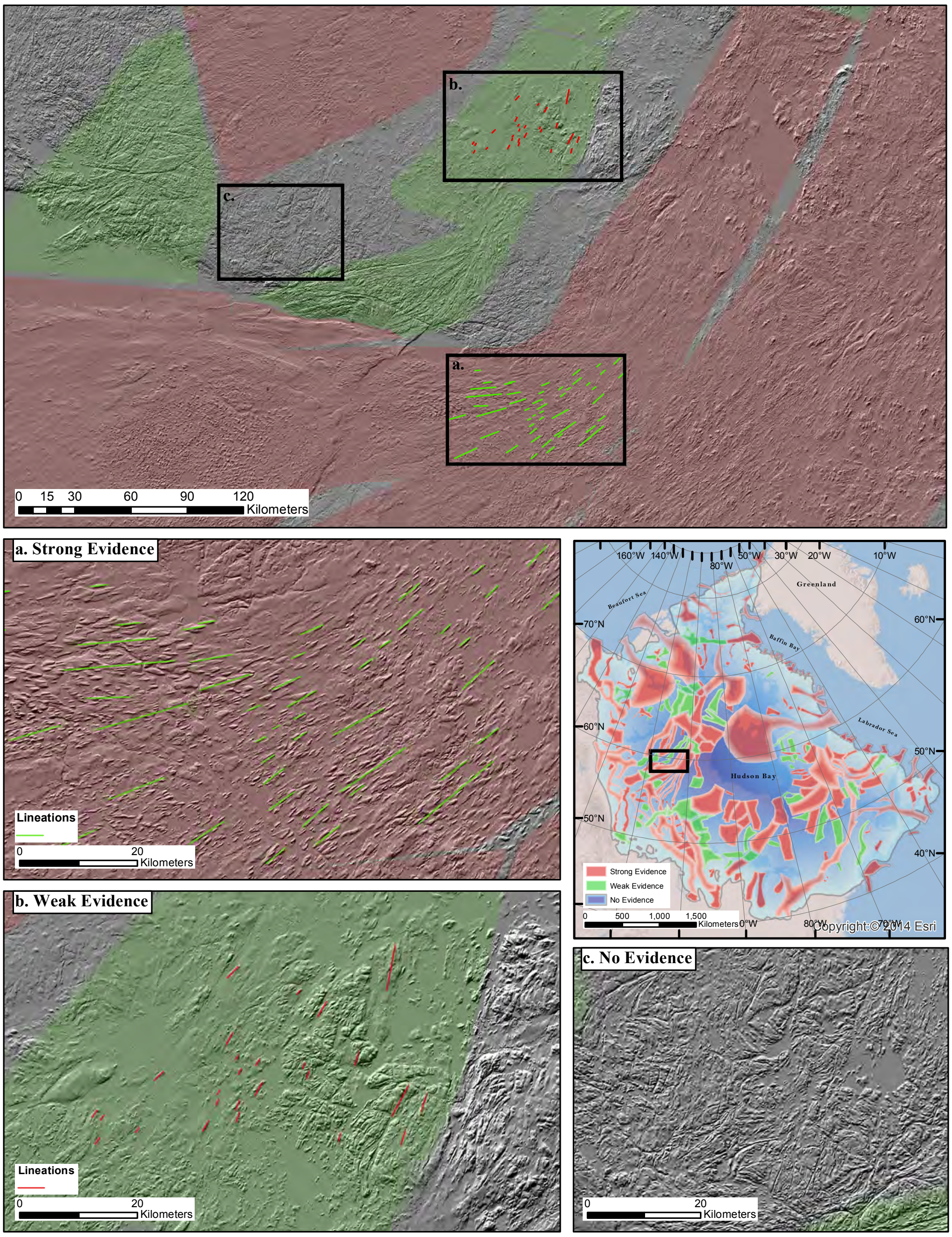

Supplementary Figure 2: Coded shaded relief maps highlighting regions with differing degrees of evidence for substantial sliding beneath a segment of the Laurentide Ice Sheet that spans the current border between Manitoba and Nunavut. Top panel shows the enlarge view of the black rectangle in the middle panel map. Middle panel (a) shows an area with strong evidence for sliding whereas the lower panels (b) and (c) show weak and no evidence for sliding, respectively.

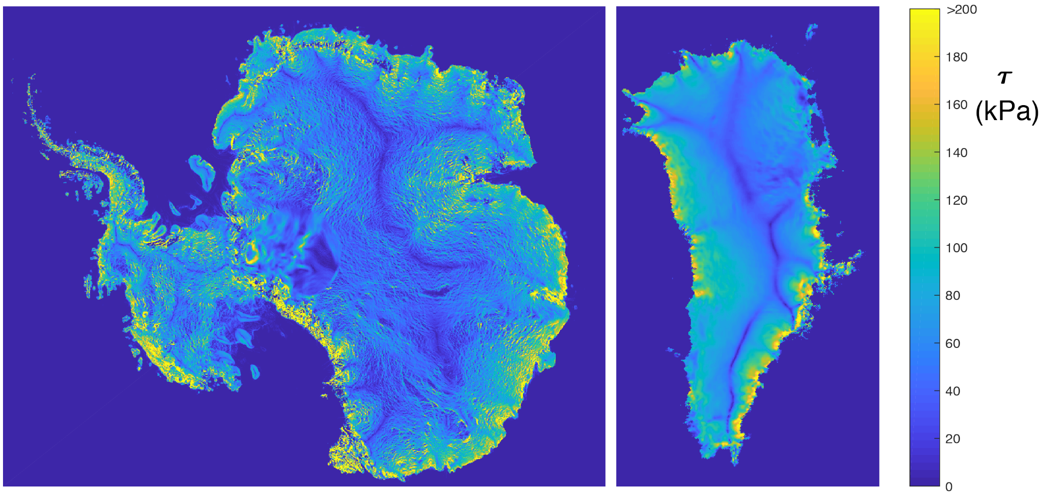

Supplementary Figure 3: Spatial distribution of driving stress τ in Antarctica[1] and Greenland[2] used to generate the cumulative distribution functions shown in Figure 1 of the main paper.

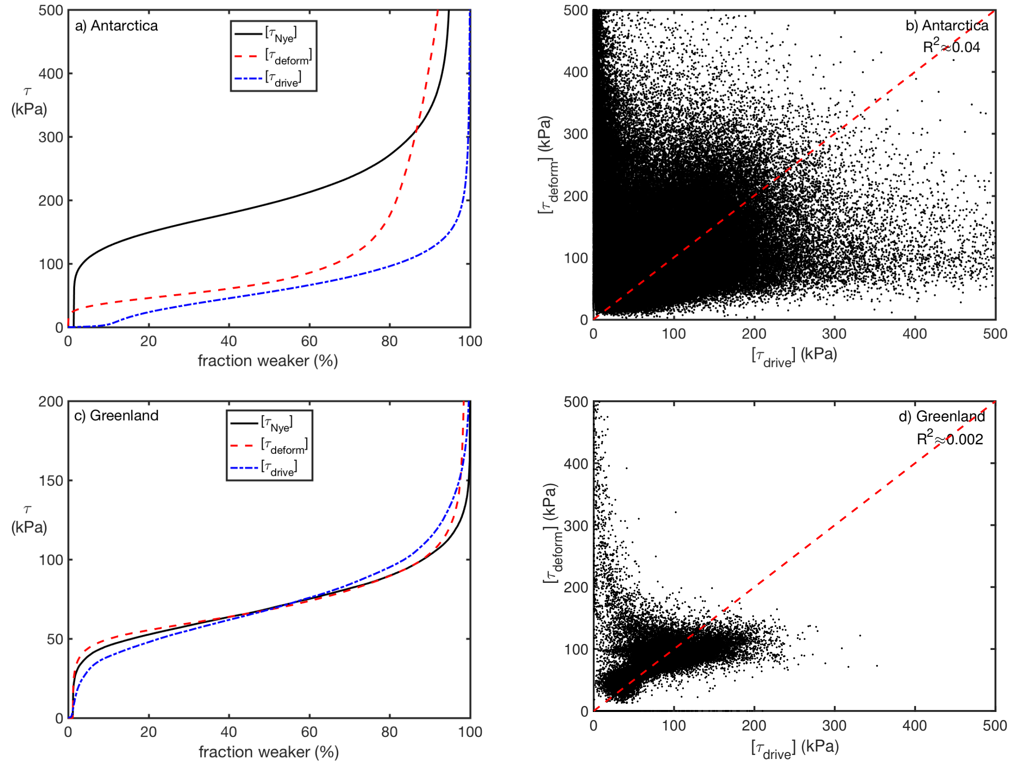

Supplementary Figure 4: Stress scales in Antarctica and Greenland. Antarctic calculations make use of surface velocities U from[3], in addition to thicknesses d and slopes $\sin\alpha$ from Fretwell et al.[1]. Greenland calculations use U from Joughin et al.[4],[5], d from Morlighem et al.[6],[7] and driving stresses τ_{drive} from MacGregor et al.[2]. a) Antarctic data. The solid black line plots the distribution implied by equation (1) of the main paper using $[\tau_{\text{Nye}}] = [Ud(\rho g \sin\alpha)^2/(2A)]^{1/(n+2)}$, where $\rho g \approx 9.0 \times 10^3\,\text{N/m}^3$ is the weight density of ice, $A \approx 2.4 \times 10^{-24}\,\text{Pa}^{-3}\text{s}^{-1}$ is ice softness, and the flow exponent is set to $n = 3$. The red dashed line gives the stress required to produce deformation that matches observed surface speeds $[\tau_{\text{deform}}] = [U/(2Ad)]^{1/n}$, and the blue dot-dashed line is the gravitational driving stress $[\tau_{\text{drive}}] = \rho g d \sin\alpha$ (shown as well in Figure 1 of the main paper). b) Antarctic data. Stress required for deformation $[\tau_{\text{deform}}]$ plotted as a function of gravitational driving stress $[\tau_{\text{drive}}]$. 58% of the data points have $[\tau_{\text{deform}}] > [\tau_{\text{drive}}]$ and so lie above the red dashed line showing the 1:1 trend. The coefficient of determination is $R^2 \approx 0.04$. c) Greenland data. Curves as in a) (note lower stress scale). d) Greenland data. Points as in b). 46% of the data points have $[\tau_{\text{deform}}] > [\tau_{\text{drive}}]$. The coefficient of determination is $R^2 \approx 0.002$.

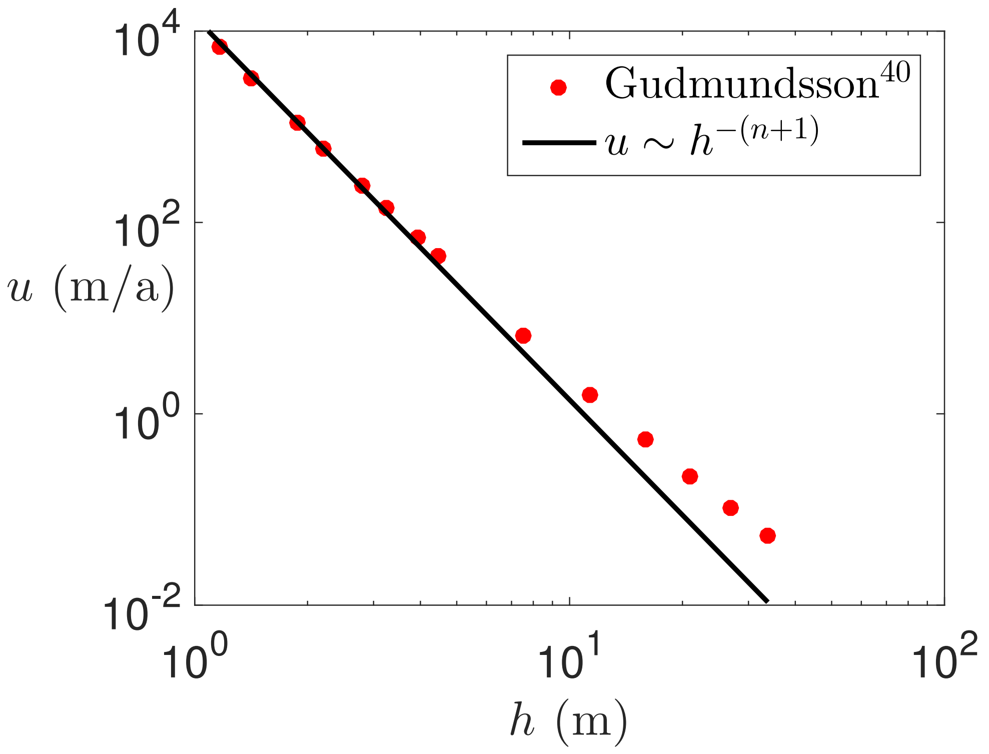

Supplementary Figure 5: Finite-element simulations by Gudmundsson[8] show that the sliding velocity u over a sinusoidal bed with amplitude h decreases as a power-law with protuberance height. As h increases the scaling breaks down, but it works sufficiently well over the entire range of glaciologically relevant sliding velocities and bump heights.

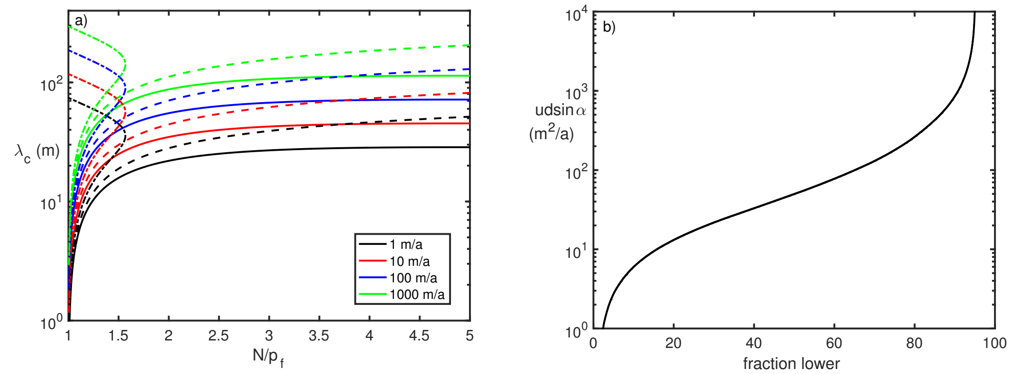

Supplementary Figure 6: a) Critical wavelength λ_c for which the drag associated with surmounting obstacles of amplitude $[h]$ at the sliding velocities $[u]$ noted in the legend matches the frictional resistance to ploughing, with $[\mu] = 0.6$, $n = 3$ and $[A] = 2.4 \times 10^{-24}\,\mathrm{Pa}^{-3}\mathrm{s}^{-1}$. The calculation of $[h]$ as a function of N/p_f is described in the methods, with the results illustrated in Figure 3 of the main paper. Solid lines depict cases with net melting at $\dot{m} = 6\,\mathrm{mm/a}$, dashed lines have $\dot{m} = 0$, and dot-dashed lines have $\dot{m} = -6\,\mathrm{mm/a}$. b) The distribution of $ud\sin\alpha$ for Antarctica obtained from ice thickness, slope[1] and velocity data[3] at 5 km resolution.

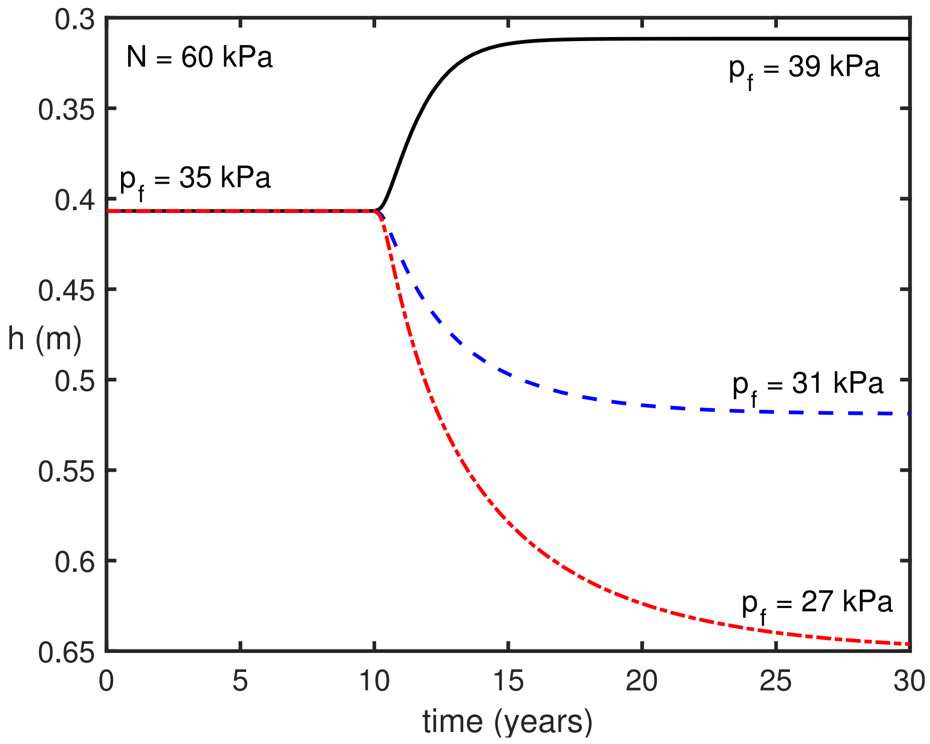

Supplementary Figure 7: Transient evolution of h in response to changes in the threshold effective stress for ice infiltration p_f. In each case $N = 60\,\text{kPa}$ is held constant and $p_f = 35\,\text{kPa}$ for the first 10 years of the simulation before an abrupt change to $p_f = 39\,\text{kPa}$ (black), 31 kPa (blue dashed), or 27 kPa (red dash-dotted). Several years are required for these adjustments, consistent with the scaling described in the text.

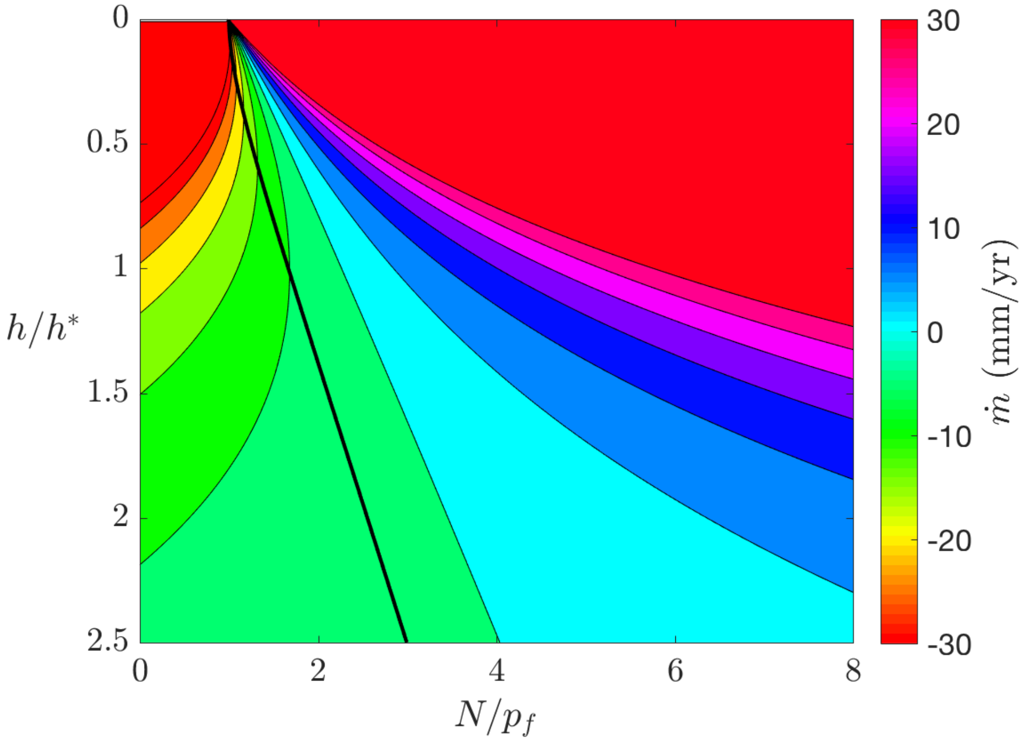

Supplementary Figure 8: Contour plot showing the steady state fringe thickness h as a function of effective pressure and meltrate. The contour lines are equally spaced (5 mm/yr increments) and represent many more examples of the curves shown in Figure 3 in the main text. The solid black line delineates the boundary between stable and unstable fringe growth. Values to the left of this solid black line are unstable.

Supplementary Note 1: Sliding Beneath Pleistocene Ice Sheets

Modern observations probe glacial behavior under contemporary environmental forcing whereas conditions during the peak of the most recent Pleistocene glacial period may have been quite different. Aspects of past glacial behavior can be deduced from evidence preserved in the sediments and landforms that were overridden, deformed, and sculpted by former ice sheets. Evans et al.[9] review the processes that lead to the generation of different types of recognizable tills, and Hambrey and Glasser[10] describe how subglacial thermal conditions can be deduced from careful analysis of these unconsolidated materials. Examples of the lateral displacement of m-thick bedrock rafts[11] have also been identified. Temporal variations in subglacial environments can complicate interpretations and produce uncertainty in assessments of the areal fractions over which sliding dominated the mass flux of former ice sheets. However, the persistence of large scale geomorphic features, including hard-bedded flow-aligned ridges sculpted by entrained debris[12,13], mega-scale glacial lineations[14,15], fields of drumlinoid sedimentary features[16,17] and cross-flow ribbed moraines[18,19] are all ascribed to sliding over relatively long durations – though the precise genetic origins of many of these features remain the subject of vigorous debate. Analyses of the distributions of such features support the conclusion that sliding conditions prevailed beneath substantial proportions of the British, Fennoscandian and Laurentide ice sheets[20,21,22].

Supplementary Figure 1 shows a compilation of regions with differing degrees of geomorphic evidence for substantial sliding at the last glacial maximum beneath the Laurentide Ice Sheet; close-ups of example regions are shown in Supplementary Figure 2, and the associated ArcMap polygons and related digital materials accompany this Supplementary Information as a zip file. Digital elevation models were obtained from the Government of Canada[23] and the United States Shuttle Radar Topography Mission (SRTM), as extracted from the OpenTopography[24] and the SRTM[25] websites see also[26]. The Laurentide Ice Sheet extent at approximately 16 ka was taken from[27], and the ESRI Tile Layer[28] was used for the shaded relief basemap. The western boundary of the Laurentide adjacent to the Cordilleran ice sheet was designated as running along the eastern side of the Rocky Mountains. The boundary between the Laurentide and Greenland Ice Sheets was assigned on the Greenland side of the intervening channel.

Digital elevation models were used to generate images of the terrain and expose potential glacial bedforms. SRTM DEMS at 30 meters resolution were used for the southernmost extent of the Laurentide. Canadian data was ex-

tracted from Government of Canada grids numbered 002 to 117, with most of these further separated into 4 by 4 grids listed from A through P at variable resolution ranging up to 70 meters. All of these DEMS were imported into ArcMap to create a mosaic dataset that could be displayed as a continuous single image. The combined mosaic dataset was then processed using the Hillshade Tool in order to show the resolution of the smaller-scale glacial features. The larger glacial troughs are easily seen without the hillshades, but finer details are visible only with the hillshades (z-factors were assigned for each DEM based on interpolation from reference values of: 1.171×10^{-5} m for 40° latitude, 1.395×10^{-5} m for 50° latitude, 1.792×10^{-5} m for 60° latitude, 2.619×10^{-5} m for 70° latitude, 5.156×10^{-5} m for 80° latitude). After processing of the mosaic datasets, the hillshades were removed and then placed again into ArcMap in order to build pyramids for the raster and improve performance.

The majority of the regions designated in Supplementary Figures 1 and 2 as exhibiting strong evidence for sliding are those identified previously as paleo ice-streams in shapefiles provided by Margold et al.[29]. We modified several of these shapefiles to both improve the correspondence with the images displayed in the figures shown by Margold et al.[29], and to extend them into regions where the Canadian DEMS show glacial landforms extending further (our "Strong Evidence" category comprises 41.7% of the Laurentide area, whereas Margold et al.[29] state that 29.6% of the area hosted ice streams). The evidence for sliding used to compile Supplementary Figures 1 and 2 is mainly in the form of drumlinoid features and lineations from the bedform imprint. Rough, mountainous surfaces typically correspond with areas where evidence of sliding is least likely to be found using our mapping techniques.

The areas we designated as bearing "Strong Evidence" for sliding at the last glacial maximum (see Supplementary Figure 2a) include well developed and preserved drumlinoid features and/or lineations that are continuous. These features are interpreted to provide strong evidence for the presence of former ice streams beneath the Laurentide Ice Sheet[29]. Together, these features blanket an area of approximately 5.36 million km^2.

The areas we designated as bearing "Weak Evidence" for sliding (see Supplementary Figure 2b) follow similar criteria, but often the lineations are either not well preserved or discontinuous. Some areas were also included in this designation when polygons were used to join two ice streams that were inferred to be connected during one time period. Ice streams that appear to intersect each other could not have both been flowing at the same time and therefore in such cases, one of the ice streams was included as strong evidence and the other as weak evidence for sliding during the LGM. The area designated as bearing "Weak Evidence" in Supplementary Figure 1 covers

approximately 1.08 million km^2.

The areas we designated as bearing "No Evidence" for sliding (see Supplementary Figure 2c) include all of the areas that show no glacial lineations or drumlinoid features. Often the surfaces in these areas are mountainous or have rough topography. While some areas of sliding evidence are located in mountainous regions, they often are accompanied with lineations showing flow directions. The area shown as having "No Evidence" for sliding in Supplementary Figure 1 occupies about 50% of the total extent of the Laurentide during the LGM. This is comparable to the areal fractions of the modern Antarctic and Greenland ice sheets that are inferred to be dominated by ice deformation rather than sliding.

A zip file that contains each of the polygons shown in Supplementary Figure 1 and the associated ArcMap data files is available for download with this Supplementary Information; red polygons are used for areas of strong evidence, green polygons are used for areas of weak evidence, blue areas are areas where our mapping techniques do not reveal evidence of sliding.

Supplementary Note 2: Basal Conditions Beneath Contemporary Ice Sheets

The ice sheet data summarized above and in Figure 1 of the main text give the driving stress averaged over length scales that are comparable to the ice thickness. Supplementary Figure 3 shows the spatial distribution of average driving stress across Antarctica and Greenland. The average basal stress is generally expected to correspond closely with the average driving stress[30]. However, beneath regions of particularly fast glacier flow the basal stress can be decreased substantially as a result of lateral stress transmission to adjacent, more slowly flowing regions, which must then be characterized by correspondingly higher basal stresses. Inverse methods have been used to account for such effects and map inferred basal stress over entire ice sheets[31,30] and at higher resolution in regions of particular interest[32,33,34]. Though detailed patterns of stress variations depend on modeling choices that are debated in the literature, the conclusion that the basal strength in sliding regions appears limited by a threshold that is on the order of 10^5 Pa (1 bar) is consistent with this actively developing body of work.

Supplementary Figure 4 uses contemporary observations of surface velocities[3,4,5], alongside surface slopes and ice thicknesses[1,6,7] and smoothed Greenland driving stress calculations[2] to compare implied stress scales over Antarctica and Greenland. In Antarctica, the stress required for ice to deform and match observed surface speeds (red dashed line in 4a and ordinate

in 4b) tends to be somewhat higher than the gravitational driving stress (blue dashed line in 4a and abscissa in 4b). The effects of sliding are a primary cause of this discrepancy, since slip at the bed causes surface velocities to be higher than the velocities obtained by deformation alone, which leads to calculated values of $[\tau_{\rm deform}]$ that are elevated relative to $[\tau_{\rm drive}]$ (i.e. points that lie above the red dashed line in 4b). The role of impurities, ice fabric and small quantities of melt water in "enhancing" ice deformation can also play a role, whereas deformation at colder temperatures is expected to take place at an ice softness A that is reduced below the temperate value used for these calculations (i.e. producing points that fall below the red dashed line in 4b). The solid black line in Supplementary Figure 4a) shows the stress scaling from equation (1) of the main paper that is implied when all ice motion occurs by deformation. The median value of $[\tau_{\rm Nye}] \approx 200\,{\rm kPa}$ is considerably higher than the observed median driving stress of $[\tau_{\rm drive}] \approx 60\,{\rm kPa}$ in Antarctica. Supplementary Figure 4c) reveals that in Greenland the distributions of driving stress, the stress required for ice deformation at $0\,^{\circ}{\rm C}$ to produce observed surface velocities, and the stress scaling from equation (1) of the main paper are remarkably similar. Note that by adopting a uniform value of A that is appropriate for temperate ice, we are displaying conservative estimates for $[\tau_{\rm Nye}]$ and $[\tau_{\rm deform}]$. If instead, a nominal temperature of $-5\,^{\circ}{\rm C}$ were used to assign A, the median values of $[\tau_{\rm Nye}]$ and $[\tau_{\rm deform}]$ would increase to 83 kPa and 94 kPa respectively, and a $-10\,^{\circ}{\rm C}$ value for A would increase them further to 100 kPa and 130 kPa. Supplementary Figure 4d) shows a direct comparison between $[\tau_{\rm deform}]$ and $[\tau_{\rm drive}]$ averaged over each 5 km by 5 km segment of the Greenlandic ice sheet. The 1:1 trend shown by the dashed red line divides the data points roughly equally, but the low coefficient of determination does not support a causal connection between these two fields. Locations with $\tau_{\rm deform}$ much greater than $\tau_{\rm drive}$ represent areas with rapid sliding.

Models of subglacial drainage networks have been used to infer distributions of effective stress that are consistent with borehole data and water transport constraints, particularly in locations with significant inputs from surface melt[35,36,37]. This approach typically predicts water pressures that are close to flotation e.g., see[37] table 2, but with considerable short-duration (daily, hourly) variability and transient values of effective stress that can exceed several MPa. (In an alternative approach, subglacial water pressures are sometimes taken to be in hydrostatic equilibrium with coastal waters so that effective stress is assumed proportional to height above buoyancy[38,39]; this leads to predictions for N that vastly exceed sparse direct observations as well as the results of hydrological studies and are therefore not considered reliable.) The discussion in the main paper appeals to steady state values of

infiltration depth h that are expected to be close to those that result from the temporally averaged effective stress. Transient calculations for the evolution of h in response to changes in effective stress[40] support the expectation that the liquid pressure distribution in the ice-infiltrated zone responds rapidly to abrupt variations in liquid pressure at the deepest extent of pore ice (changing the melt rate $\dot{m}$ and satisfying the vertical force balance without significantly altering the contribution transmitted to the underlying sediment contacts), whereas h evolves much more slowly (see Section 4 below).

Supplementary Note 3: The Effects of Heterogeneous Sediment Entrainment on Bed Strength

Considerable uncertainty persists in our understanding of the "sliding law" that governs how the bed strength τ varies with the slip rate u and other controlling variables (e.g. melt rate, basal topography, hydrological parameters) that describe the local subglacial environment. To make progress, the scaling displayed in equation (2) of the main paper proceeds from a highly idealized analysis of bed processes. The magnitude of the predicted $[\tau]$ and the likely effects of additional complexities deserve further consideration.

In the simplest scenario with sliding over an unconsolidated porous half-space, increases to the effective stress N beyond the threshold for ice infiltration p_f are expected to lead initially to frictional ploughing of weak liquid-saturated sediments by ice-infiltrated basal keels. For characteristic friction coefficient $[\mu]$, the characteristic or average bed strength in this regime can be written as $[\tau] = [\mu][N]$, with $[N]$ representing a characteristic effective stress (defined as the spatially averaged effective normal stress borne by particle contacts on the slip surface). The finite-element calculations by Gudmundsson[8] shown in Supplementary Figure 5 confirm earlier analytical predictions[41,42] that the shear stress τ required to surmount obstacles of amplitude h and wavelength λ for a viscous power-law fluid, as described by Glen's law, is

$$\tau \sim 2C\left(\frac{h}{\lambda}\right)\left[\frac{uh}{A\lambda^2}\right]^{1/n}, \tag{1}$$

where A is the softness parameter, n is the rheological exponent, u is the sliding velocity, and the constant $C \approx 30$ accounts for geometrical details. Recognizing that heterogeneous basal conditions are ubiquitous and assuming that the amplitude of topographic irregularities on the basal slip surface scales with the characteristic depth of ice infiltration $[h]$, we expect ploughing to be circumvented by localized deformation over obstacles with characteris-

tic wavelengths of

$$[\lambda] \geq \lambda_c = \left\{ \frac{[2C]^n [h]^{n+1} [u]}{[\mu]^n [N]^n [A]} \right\}^{1/(n+2)} . \quad (2)$$

Supplementary Figure 6a) shows predicted values of λ_c as a function of N/p_f when $[h]$ follows the behavior illustrated in Figure 3 of the main paper. While the actual distribution of h at the glacial bed depends on details of the local variations in sediment properties, effective stress, and heat balance, these calculations support the expectation that for glaciologically relevant sliding speeds the resistance to slip associated with heterogeneous ice infiltration and either frictional ploughing for obstacles smaller than λ_c or drag due to deformation over obstacles larger than λ_c is limited to a small multiple of p_f. Modern observations from Antarctica can be used to estimate glaciologically relevant values of the product $[a][l][\sin\alpha]$ for use in the scaling law represented by equation (2) of the main paper. Supplementary Figure 6b) shows the distribution of $ud \sin\alpha$ obtained from the ice geometry of Bedmap2[1] and the surface velocities of Rignot et al.[3]. Because surface velocities combine both deformation through the ice column and sliding at the bed, Supplementary Figure 6b) gives an upper bound on modern values of $[u][d][\sin\alpha]$ in Antarctica. Assuming that Antarctic velocities are approximately in balance with upstream accumulation implies that $[a][l] \approx [u][d]$ and the distribution shown in Supplementary Figure 6b) can be interpreted as roughly equivalent to that of $[a][l][\sin\alpha]$. For typical combinations of the relevant parameters, equation (2) of the main paper predicts characteristic values of $[\tau]$ that are comparable to the range of observed driving stresses on contemporary ice sheets and mountain glaciers.

To illustrate the resistance to deformation over obstacles we have used the recommended value of softness A reported by[43] for ice near its pressure melting point. Moore[44] provides an illuminating review highlighting the uncertainties that persist concerning the rheology of debris-rich ice, and the contradictory evidence for both softening and stiffening in comparison with uncontaminated ice. Extensions to our work would certainly benefit from an improved understanding of these issues. However, the weak dependence on A shared by the expressions for λ_c in supplementary equation (2) and for $[\tau]$ in equation (2) of the main paper provide confidence that the implementation of more complicated and realistic rheological treatments would be unlikely to have an appreciable affect on essential aspects of the behavior that we predict. Softer (stiffer) basal ice would tend to lower (increase) the threshold wavelength λ_c for the transition from ploughing to deformation over obstacles of a given characteristic height. For example, increasing A by a factor of 20–40, as inferred by Pettit et al.[45] for the debris-rich basal ice beneath Taylor

Glacier in Antarctica, would lower λ_c by approximately a factor of 2. For a fixed characteristic obstacle spacing λ, the scaling law from equation (2) of the main paper predicts a decrease in $[\tau]$ by up to a factor of 2.5 when A is softened 20–40 fold. In contrast, empirical and laboratory evidence[44] for decreased A with added debris content would suggest somewhat larger values of λ_c and $[\tau]$ than those that we have calculated. Nevertheless, even with such complications the inference that $[\tau]$ and $[p_f]$ are similar in size remains unchanged.

Supplementary Note 4: Transient ice infiltration

Modern observations and theoretical considerations motivate increasingly sophisticated efforts to forecast the response of glaciers and ice sheets to changing climate. Recalling the description of force balance given in equation (3) of the main paper, even independent of transient modifications to conditions on the boundaries of an ice sheet, glacial sliding over large distances causes basal ice to encounter variations in the characteristics of the underlying geological materials (gauged by p_f, T_f, ϕ, $\Delta\rho_{\text{till}}$, S_i and k), the effective stress (N) and the heat flux environment ($\dot{m}$ and G). With these considerations in mind, the time scale over which changes in ice infiltration depth h can take place and affect basal strength is clearly relevant for assessments of how sliding resistance evolves.

Adjustments to h are constrained to satisfy energy and mass balance conditions in addition to the force balance condition represented by equation (3) of the main paper. A straightforward scaling argument can be used to assess the length of time required to achieve a new steady state temperature profile following a perturbation to any of the variables that control h. The latent heat associated with changing the relative proportions of ice and liquid water in the pore space is much more important than the sensible heat associated with the slight modifications to the temperature that also accompany changes in h. With this in mind, the mass and energy conservation conditions imply that the evolution in temperature T over time t within the ice-infiltrated zone can be approximated by cf.[40] Eq. B4

$$\left(-\phi\rho L\frac{\mathrm{d}S_i}{\mathrm{d}T}\right)\left(\frac{\partial T}{\partial t}-\dot{m}\frac{\partial T}{\partial z}\right)\approx\frac{\partial}{\partial z}\left(K_e\frac{\partial T}{\partial z}\right)\ , \tag{3}$$

where K_e is the effective thermal conductivity of the ice–liquid–sediment mixture.

The requirement that force balance be maintained as h gradually responds to a rapid perturbation (for example to N as fluid pressure is altered or p_f following slip onto a till characterized by coarser or finer particles) suggests

that, as the most responsive element in the system, the fluid pressure distribution throughout the ice infiltrated zone must also adjust very quickly; the constraints of mass balance and Darcy's law imply that $\dot{m}$ can undergo large swings in behavior during the adjustment process[40]. Accordingly, a conservative estimate of the characteristic time scale $[\Delta t]$ over which a new equilibrium state can be established is obtained by balancing the first term on the left with the term on the right side of supplementary equation (3) to get

$$[\Delta t] \sim \frac{[\phi]\rho L[h^*][\Delta S_i]}{[K_e][G]} \ , \tag{4}$$

where we have identified the conductive distance with $[h^*]$ and define $[\Delta S_i]$ as the characteristic size of changes in S_i, for example at the top of the ice-infiltrated zone. Assigning nominal values of $[\phi] \approx 0.4$, $[h^*] = 1\,\text{m}$, $[K_e][G] \approx 0.06\,\text{W/(m K)}$, $[\Delta S_i] \approx 0.05$, and noting that $\rho L \approx 3 \times 10^8\,\text{J/m}^3$ produces a nominal value of $[\Delta t] \approx 3\,$years, which is consistent with the results of numerical calculations (e.g. see Supplementary Figure 7). Over timescales that are much shorter than a few years, variations in basal conditions promote significant fluctuations in $\dot{m}$, but the depth of ice infiltration h is expected to reflect the multi-year averaged conditions. In contrast, gradual changes in basal conditions that take place over decades or centuries are expected to be accompanied by adjustments in the depth of ice infiltration that are comparable to a sequence of steady states; for example, like those shown in Figure 3 of the main paper or in Supplementary Figure 8 where the meltrate $\dot{m}$ is contoured.

Supplementary References

[1] P. Fretwell, H. D. Pritchard, D. G. Vaughan, J. L. Bamber, N. E. Barrand, R. Bell, C. Bianchi, R. G. Bingham, D. D. Blankenship, G. Casassa, et al. Bedmap2: improved ice bed, surface and thickness datasets for antarctica. *Cryosphere*, 7(1), 2013. doi: 10.5194/tc-7-375-2013.

[2] J. A. MacGregor, M. A. Fahnestock, G. A. Catania, A. Aschwanden, G. D. Clow, W. T. Colgan, S. P. Gogineni, M. Morlighem, S. Nowicki, J. D. Paden, et al. A synthesis of the basal thermal state of the Greenland Ice Sheet. *J. Geophys. Res.*, 121(7):1328–1350, 2016.

[3] E. Rignot, J. Mouginot, and B. Scheuchl. Ice flow of the Antarctic ice sheet. *Science*, 333(6048):1427–1430, 2011. doi: 10.1126/science.1208336.

[4] I. Joughin, B. Smith, I. Howat, T. Scambos, and T. Moon. Greenland flow variability from ice-sheet-wide velocity mapping. *Journal of Glaciology*, 56:415–430, 2010.

[5] I. Joughin, B. Smith, I. Howat, and T. Scambos. Measures greenland ice sheet velocity map from insar data, version 2. *NASA National Snow and Ice Data Center Distributed Active Archive Center, Boulder, Colorado, USA (doi: https://doi.org/10.5067/OC7B04ZM9G6Q [accessed 03/30/18])*, 2017.

[6] Mathieu Morlighem, Chris N Williams, Eric Rignot, Lu An, Jan Erik Arndt, Jonathan L Bamber, Ginny Catania, Nolwenn Chauché, Julian A Dowdeswell, Boris Dorschel, et al. IceBridge BedMachine Greenland, Version 3. Boulder Colorado USA. NASA National Snow and Ice Data Center Distributed Active Archive Center, 2017.

[7] Mathieu Morlighem, Chris N Williams, Eric Rignot, Lu An, Jan Erik Arndt, Jonathan L Bamber, Ginny Catania, Nolwenn Chauché, Julian A Dowdeswell, Boris Dorschel, et al. Bedmachine v3: Complete bed topography and ocean bathymetry mapping of greenland from multibeam echo sounding combined with mass conservation. *Geophysical Research Letters*, 44(21), 2017.

[8] G. H. Gudmundsson. Basal-flow characteristics of a non-linear flow sliding frictionless over strongly undulating bedrock. *J. Glaciol.*, 43 (143):80–89, 1997. doi: 10.1017/S0022143000002835.

[9] DJA Evans, ER Phillips, JF Hiemstra, and CA Auton. Subglacial till: formation, sedimentary characteristics and classification. *Earth-Science Reviews*, 78(1):115–176, 2006.

[10] Michael J Hambrey and Neil F Glasser. Discriminating glacier thermal and dynamic regimes in the sedimentary record. *Sedimentary Geology*, 251:1–33, 2012.

[11] John F Hiemstra, David JA Evans, and Colm O Cofaigh. The role of glacitectonic rafting and comminution in the production of subglacial tills: Examples from southwest Ireland and Antarctica. *Boreas*, 36(4): 386–399, 2007.

[12] Nick Eyles and Mike Doughty. Glacially-streamlined hard and soft beds of the paleo-Ontario ice stream in Southern Ontario and New York state. *Sedimentary Geology*, 338:51–71, 2016.

[13] Maarten Krabbendam, Nick Eyles, Niko Putkinen, Tom Bradwell, and Lina Arbelaez-Moreno. Streamlined hard beds formed by palaeo-ice streams: A review. *Sedimentary Geology*, 338:24–50, 2016.

[14] Chris D Clark. Mega-scale glacial lineations and cross-cutting ice-flow landforms. *Earth surface processes and landforms*, 18(1):1–29, 1993.

[15] Sarah L Greenwood and Johan Kleman. Glacial landforms of extreme size in the Keewatin sector of the Laurentide Ice Sheet. *Quaternary Science Reviews*, 29(15):1894–1910, 2010.

[16] Maris Rattas and Jan A Piotrowski. Influence of bedrock permeability and till grain size on the formation of the saadjärve drumlin field, estonia, under an east-baltic weichselian ice stream. *Boreas*, 32(1):167–177, 2003.

[17] Arthur S Dyke and Thomas F Morris. Drumlin fields, dispersal trains, and ice streams in arctic canada. *The Canadian Geographer/Le Géographe canadien*, 32(1):86–90, 1988.

[18] Paul Dunlop and Chris D Clark. The morphological characteristics of ribbed moraine. *Quaternary Science Reviews*, 25(13):1668–1691, 2006.

[19] Chris R Stokes, Martin Margold, and Timothy T Creyts. Ribbed bedforms on palaeo-ice stream beds resemble regular patterns of basal shear stress (traction ribs) inferred from modern ice streams. *Journal of Glaciology*, 62(234):696–713, 2016.

[20] Geoffrey Boulton and Magnus Hagdorn. Glaciology of the british isles ice sheet during the last glacial cycle: form, flow, streams and lobes. *Quaternary Science Reviews*, 25(23):3359–3390, 2006.

[21] Johan Kleman and Clas Hättestrand. Frozen-bed fennoscandian and laurentide ice sheets during the last glacial maximum. *Nature*, 402(6757): 63–66, 1999.

[22] C. R. Stokes, M. Margold, C. D. Clark, and L. Tarasov. Ice stream activity scaled to ice sheet volume during Laurentide Ice Sheet deglaciation. *Nature.*, 530(7590):322–326, 2016. doi: 10.1038/nature16947.

[23] Government of Canada. Canadian Digital Elevation Model, 2017. URL `http://open.canada.ca/data/en/dataset/7f245e4d-76c2-4caa-951a-45d1d2051333`.

[24] OpenTopography. Shuttle Radar Topography Mission (SRTM GL1) Global 30m, 2000. URL http://opentopo.sdsc.edu/raster?opentopoID=OTSRTM.082015.4326.1.

[25] SRTM. Shuttle Radar Topography Mission (SRTM GL1) Global 30m, 2000. URL https://www2.jpl.nasa.gov/srtm/index.html.

[26] Tom G Farr, Paul A Rosen, Edward Caro, Robert Crippen, Riley Duren, Scott Hensley, Michael Kobrick, Mimi Paller, Ernesto Rodriguez, Ladislav Roth, et al. The shuttle radar topography mission. *Reviews of Geophysics*, 45(2), 2007.

[27] Arthur S Dyke, Andrew Moore, and Louis Robertson. *Deglaciation of North America*. Geological Survey of Canada Ottawa, ON, 2003.

[28] ESRI Tile Layer. World_Shaded_Relief (MapServer), 2014. URL https://services.arcgisonline.com/ArcGIS/rest/services.

[29] M. Margold, C. R. Stokes, and C. D. Clark. Ice streams in the laurentide ice sheet: Identification, characteristics and comparison to modern ice sheets. *Earth Sci. Rev.*, 143:117 – 146, 2015. ISSN 0012-8252. doi: 10.1016/j.earscirev.2015.01.011.

[30] M. Morlighem, H. Seroussi, E. Larour, and E. Rignot. Inversion of basal friction in Antarctica using exact and incomplete adjoints of a higher-order model. *J. Geophys. Res.*, 118(3):1746–1753, 2013. doi: 10.1002/jgrf.20125.

[31] D. Pollard and R. M. DeConto. A simple inverse method for the distribution of basal sliding coefficients under ice sheets, applied to Antarctica. *Cryosphere*, 6(5):953, 2012. doi: 10.5194/tcd-6-1405-2012.

[32] O. V. Sergienko, T. T. Creyts, and R. C. A. Hindmarsh. Similarity of organized patterns in driving and basal stresses of Antarctic and Greenland ice sheets beneath extensive areas of basal sliding. *Geophys. Res. Lett.*, 2014. doi: 10.1002/2014GL059976.

[33] Daniel R Shapero, Ian R Joughin, Kristin Poinar, Mathieu Morlighem, and Fabien Gillet-Chaulet. Basal resistance for three of the largest greenland outlet glaciers. *J. Geophys. Res.*, 121(1):168–180, 2016.

[34] Brent Minchew, Mark Simons, Helgi Björnsson, Finnur Pálsson, Mathieu Morlighem, Helene Seroussi, Eric Larour, and Scott Hensley. Plastic bed beneath hofsjökull ice cap, central iceland, and the sensitivity of ice

flow to surface meltwater flux. *Journal of Glaciology*, 62(231):147–158, 2016.

[35] Winnie Chu, Timothy T Creyts, and Robin E Bell. Rerouting of subglacial water flow between neighboring glaciers in West Greenland. *J. Geophys. Res.*, 121(5):925–938, 2016.

[36] Basile Fleurian, Mathieu Morlighem, Helene Seroussi, Eric Rignot, Michiel R Broeke, Peter Kuipers Munneke, Jeremie Mouginot, Paul CJP Smeets, and Andrew J Tedstone. A modeling study of the effect of runoff variability on the effective pressure beneath Russell Glacier, West Greenland. *J. Geophys. Res.*, 121(10):1834–1848, 2016.

[37] Patrick J Wright, Joel T Harper, Neil F Humphrey, and Toby W Meierbachtol. Measured basal water pressure variability of the western Greenland Ice Sheet: Implications for hydraulic potential. *J. Geophys. Res.*, 121(6):1134–1147, 2016.

[38] F. M. Nick, A. Vieli, M. L. Andersen, I. Joughin, A. Payne, T. L. Edwards, F. Pattyn, and R. S. W. van de Wal. Future sea-level rise from Greenland?s main outlet glaciers in a warming climate. *Nature*, 497 (7448):235, 2013. doi: 10.1038/nature12068.

[39] L. A. Stearns and C. J. van der Veen. Friction at the bed does not control fast glacier flow. *Science*, 2018. ISSN 0036-8075. doi: 10.1126/science.aat2217.

[40] A. W. Rempel. A theory for ice-till interactions and sediment entrainment beneath glaciers. *J. Geophys. Res.*, 113(F1), 2008. ISSN 2156-2202. doi: 10.1029/2007JF000870. F01013.

[41] J. Weertman. On the sliding of glaciers. *J. Glaciol*, 3(21):33–38, 1957. doi: 10.3198/1957JoG3-21-33-38.

[42] B. Kamb. Sliding motion of glaciers: theory and observation. *Rev. Geophys.*, 8(4):673–728, 1970. doi: 10.1029/RG008i004p00673.

[43] K. M. Cuffey and W. S. B. Paterson. *The Physics of Glaciers (Fourth Edition)*. ISBN 9780123694614. Elsevier, 2010.

[44] Peter L Moore. Deformation of debris-ice mixtures. *Rev. Geophys.*, 52 (3):435–467, 2014.

[45] Erin C Pettit, Erin N Whorton, Edwin D Waddington, and Ronald S Sletten. Influence of debris-rich basal ice on flow of a polar glacier. *Journal of Glaciology*, 60(223):989–1006, 2014.
